# Extracellular vesicles from *Paracoccidioides brasiliensis* induced M1 polarization *in vitro*

**DOI:** 10.1038/srep35867

**Published:** 2016-10-24

**Authors:** Thiago Aparecido da Silva, Maria Cristina Roque-Barreira, Arturo Casadevall, Fausto Almeida

**Affiliations:** 1 Ribeirao Preto Medical School, Department of Cellular and Molecular Biology, University of Sao Paulo, Ribeirao Preto, SP, Brazil; 2 Department of Molecular Microbiology and Immunology, Johns Hopkins Bloomberg School of Public Health Baltimore, MD, USA

## Abstract

Extracellular vesicles (EVs) released by eukaryotes, archaea, and bacteria contain proteins, lipids, polysaccharides, and other molecules. The cargo analysis of EVs shows that they contain virulence factors suggesting a role in the pathogenesis of infection. The proteome, lipidome, RNA content, and carbohydrate composition of EVs from *Paracoccidioides brasiliensis* and *Paracoccidioides lutzii* were characterized. However, the effects of *P. brasiliensis* EVs on the host immune system have not yet been investigated. Herein, we verified that EVs from *P. brasiliensis* induce the production of proinflammatory mediators by murine macrophages in a dose-dependent manner. Addition of EV to macrophages also promoted transcription of the M1-polarization marker iNOs and diminish that of the M2 markers Arginase-1, Ym-1, and FIZZ-1. Furthermore, the augmented expression of M2-polarization markers, stimulated by IL-4 plus IL-10, was reverted toward an M1 phenotype in response to secondary stimulation with EVs from *P. brasiliensis*. The ability of EVs from *P. brasiliensis* to promote M1 polarization macrophages favoring an enhanced fungicidal activity, demonstrated by the decreased CFU recovery of internalized yeasts, with comparable phagocytic efficacy. Our results suggest that EVs from *P. brasiliensis* can modulate the innate immune response and affect the relationship between *P. brasiliensis* and host immune cells.

*Paracoccidioides brasiliensis* and *Paracoccidioides lutzii* are thermodimorphic pathogenic fungi that are the etiologic agents of paracoccidioidomycosis (PCM), a disease endemic to Latin America associated with severe public health problems. PCM starts by the inhalation of airborne propagules from fungal mycelia, which convert into yeasts in the lungs, causing granuloma formation and adversely affecting the functions of other infected organs^1^,^2^,^3^. Active PCM mostly affects the lungs, but it may develop into an acute form or, more frequently, reactivate later as a chronic and insidious disease that can disseminate to many different organs and tissues^4^,^5^,^6^. Although the mechanisms by which the host controls PCM remain unclear, modulation of the immune system is crucial to determine the progress of the infection, and cellular immunity is the principal defense mechanism involved in PCM.

The secretion of fungal components is critical for the establishment of a successful infection, as is reported for *Candida albicans*^7^, *Histoplasma capsulatum*^8^, *Cryptococcus neoformans*^9^. Some fungal components are secreted through conventional mechanisms, involving vesicles of the post-Golgi network that move to and fuse with the plasma membrane to release their cargo through exocytosis^10^. Eukaryotes evolved unconventional secretion mechanisms to release proteins into the extracellular milieu^11^,^12^,^13^,^14^,^15^, including secretion through exosomes. Exosomes are formed by internal vesicles present in the lumen of endosomes, which generate multivesicular bodies that fuse with the plasma membrane, resulting in the release of internal vesicles into the extracellular milieu. These fungal extracellular vesicles (EVs), also termed fungal exosomes, contain a wide assortment of proteins, lipids, polysaccharides, and nucleic acid. In the case of *P. brasiliensis*, EVs were first purified from the supernatant of yeast cultures^16^ and identified by the presence of terminal Gal-α –(1,3)-Gal (α –Gal) epitopes. Subsequently, EVs from *P. brasiliensis* and *P. lutzii* were characterized with regards to their proteome^17^, lipidome^18^, RNA content^19^, and carbohydrate composition^20^.

Several virulence factors that enhance pathogen survival in the host by allowing evasion of immune surveillance possible have been associated with fungal EVs^21^,^22^. In addition, several cellular factors released by fungi have been associated with immunomodulation^14^,^15^,^23^,^24^,^25^,^26^,^27^. Studies on the immunomodulatory activity of EVs allow a better understanding of how the host immune response may be influenced by fungal components. Immunomodulation by EVs may be directed toward macrophages, which are essential in guiding the ensuing development of adaptive immunity^28^. It is known that macrophages can be engaged in different types of activation: “classic” M1 macrophages are inflammatory cells that phagocytize and kill microbes, while “alternative” M2 cells work in favor of angiogenesis, and tissue remodeling and repair^29^. However, capacity for EVs from *P. brasiliensis* to mediate immunomodulatory effects has not been investigated. A recent study showed that in chronic PCM the majority of macrophages are M2^30^, which contribute to the development of a Th2 immune response that is associated with susceptibility to the infection^30^,^31^. Therefore, we hypothesized that EVs from *P. brasiliensis* could play a determinant role in the interaction of the pathogen with macrophages and could modulate host immunity, as was reported for EVs from *C. neoformans*^15^.

In this study, we investigated whether EVs from *P. brasiliensis* affected the polarization of macrophages and their effects on macrophage phagocytosis and fungicidal activity against *P. brasiliensis*. EVs from *P. brasiliensis* were found to induce M1 polarization of macrophages and enhance the fungicidal activity of murine macrophages. Our results suggest that EVs of *P. brasiliensis* can influence the interaction of the fungus with host cells.

## Results

### *P. brasiliensis* EVs induce proinflammatory mediators in murine peritoneal macrophages

**Figure 1:**
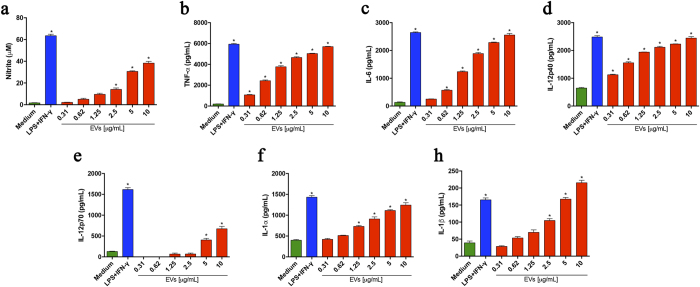
EVs from *P. brasiliensis* induce the production of proinflammatory mediators by peritoneal macrophages. Peritoneal macrophages (1.5 × 10^6^/mL) of C57BL/6 mice were incubated at 37 °C for 48 h with the indicated amounts of extracellular vesicles from *P. brasiliensis* (x axis). The medium and LPS (1 μg/mL) plus IFN-γ (2 ng/mL) were used as negative and positive controls, respectively. The culture supernatants were assessed for nitrite (**a**), TNF-α (**b**), IL-6 (**c**), IL-12p40 (**d**), IL-12p70 (**e**), IL-1α (**f**), and IL-1β (**g**) concentrations. The results, expressed as mean ± SEM, were compared to the levels in unstimulated cells (with the medium only). Differences were considered significant when p < 0.05 (*).

Extracellular vesicles have recently been isolated from culture supernatants of *P. brasiliensis* yeasts and characterized regarding their proteome, lipidome, and carbohydrate content^18^,^20^. Since the influence of the vesicles on the host innate immunity cells has not been evaluated, we examined whether EVs produced by *P. brasiliensis* yeasts could stimulate macrophages. To do so we added various quantities of EVs to murine peritoneal macrophages and assessed the culture supernatant for the production of nitric oxide and cytokines after 48 h of incubation. EVs induced the release of NO, IL-12p40, IL-12p70, IL-6, TNF-α, IL-1α, and IL-1β in a dose-dependent manner (Fig. 1). In contrast, the detected levels of IL-10 were as low as those released by unstimulated macrophages (data not show). These findings show that EVs induce murine macrophages to produce proinflammatory mediators.

### *P. brasiliensis* EVs stimulate J774A.1 cells to produce proinflammatory cytokines

**Figure 2:**
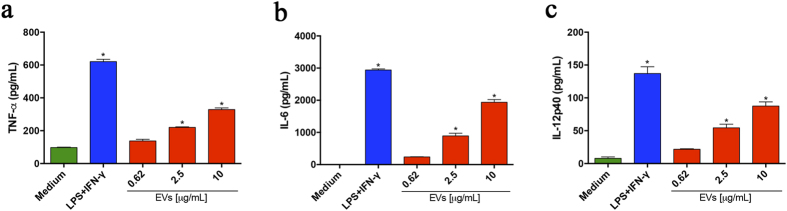
EVs from *P. brasiliensis* induce the production of proinflammatory mediators by the macrophage cell line J774A.1. J774A.1 cells (1.5 × 10^6^/mL) were incubated at 37 °C for 48 h with the indicated amounts of extracellular vesicles from *P. brasiliensis* (x axis). The medium and LPS (1 μg/mL) plus IFN-γ (2 ng/mL) were used as negative and positive controls, respectively. The culture supernatants were assessed for TNF-α (**a**), IL-6 (**b**), and IL-12p40 (**c**) levels. The results, expressed as mean ± SEM, were compared to the levels in unstimulated cells (with the medium only). Differences were considered significant when p < 0.05 (*).

We also evaluated the effects of the EVs on murine macrophages of the cell line J774A.1. The cells were incubated for 48 h with several quantities of EVs, and the culture supernatants were assessed for cytokines production. TNF-α, IL-6, and IL-12 levels significantly increased in response to the EV stimulus in a dose-dependent manner (Fig. 2). These observations corroborate with the capacity of EVs to promote a proinflammatory profile in murine macrophages.

### *P. brasiliensis* EVs induce macrophage M1 phenotype

**Figure 3:**
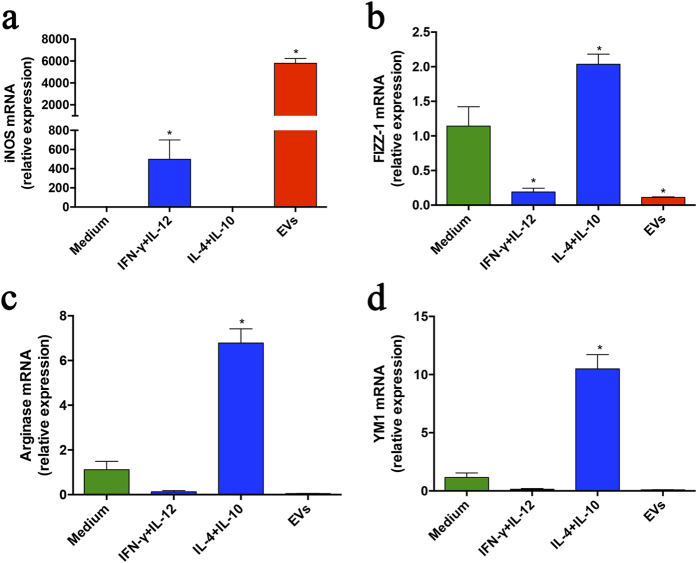
EVs from *P. brasiliensis* promote macrophage classical activation. Macrophages (2 × 10^6^/mL) of C57BL/6 mice were incubated at 37 °C for 6 h with EVs (10 *μ*g/mL) and IFN-γ (2 ng/mL) plus IL-12p40 (50 ng/mL) as M1 inducers (classical activation) or IL-10 plus IL-4 (50 ng/mL both) as M2 inducers (alternative activation). The medium was used as a negative control. Following RNA extraction and conversion into cDNA, the relative expressions of iNOS (**a**), FIZZ1 (**b**), Arginase-1 (**c**), and Ym-1 (**d**) were determined by real-time PCR. The results, expressed as mean ± SEM, were compared to those obtained from the negative control. Differences were considered significant when p < 0.05 (*).

The high production of proinflammatory mediators suggests that EVs could favor the development of macrophages toward the “classical” M1 activation phenotype. To investigate this hypothesis, we extracted total RNA of macrophages that had been incubated with EVs for 6 h and carried out the relative quantification of transcripts of M1 (iNOs) and M2 (Arginase-1, Ym-1, and FIZZ-1) polarization markers. The iNOs mRNA increased 5800-fold in EV-stimulated murine peritoneal macrophages, a response that overcame that induced by the IFN-γ plus IL-12 stimulus (Fig. 3a). In contrast, the relative expression of FIZZ-1 mRNA was reduced significantly under the EV stimulus. Consistently, the expression of Arginase-1 and Ym-1 mRNA in the presence of vesicles remained close to that found in unstimulated cells (Fig. 3b–d). These results indicate that *P. brasiliensis* EVs promote the polarization of macrophages towards the “classical” M1 phenotype.

### EVs from *P. brasiliensis* induce switching from M2 to M1 macrophages

**Figure 4:**
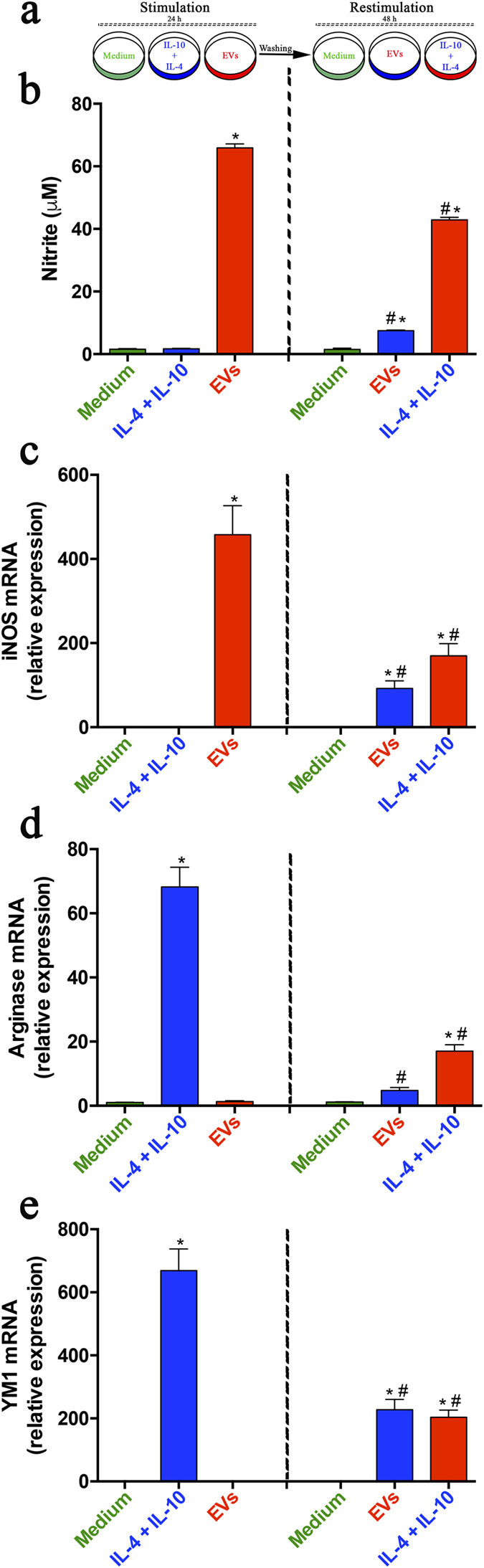
EVs from *P. brasiliensis* induce switching from M2 to M1 macrophages. **(a)** Peritoneal macrophages (2 × 10^6^/mL) were incubated at 37 °C with EVs (10 *μ*g/mL; red), IL-4 plus IL-10 (50 ng/mL both; blue), and the medium as a negative control (green). After 24 h, the cells were washed and subjected to re-stimulation for 48 h as follows: the macrophages that were stimulated with IL-4 plus IL-10 (blue) during the first 24 hours were then re-stimulated with EVs (red); those stimulated for 24 h with EVs (red) were then re-stimulated with IL-4 plus IL-10 (blue). The culture supernatants were assessed for nitrite concentration (**b**), and cells were analyzed by real-time PCR for the relative expression of iNOS (**c**), Arginase-1 (**d**), and Ym-1 (**e**). The results, expressed as mean ± SEM, were compared to those obtained from the negative control. Differences were considered significant when p < 0.05 (*). An additional comparison was made between the results obtained at 24 and 48 h, represented as blue or red bars (#). Differences were considered significant when p < 0.05.

In fungal infections, macrophage polarization is plastic, the cells being able to change from the M2 (‘non-protective’) to the M1 (‘protective’) phenotype^32^. We investigated whether EVs from *P. brasiliensis* could promote the switching of macrophages from an M2 to an M1 phenotype. We stimulated murine peritoneal macrophages with EVs (10 μg/mL) or IL-4 (50 ng/mL) plus IL-10 (50 ng/mL) to develop M1 or M2 macrophages, respectively. The medium alone was used as a negative control. After 24 h, the cells were washed and subjected to a secondary stimulation for 48 h: cells that had previously been stimulated with IL-4 plus IL-10 were now re-stimulated with EVs, and vice-versa. Cell supernatants were assessed for NO concentration. We found that the NO production by cells stimulated with IL-4 plus IL-10 was low and was reverted by EVs re-stimulation (Fig. 4b), whereas the NO production by macrophages stimulated with EVs was high and was significantly decreased when re-stimulated with IL-4 plus IL-10 (Fig. 4b). The relative expression of M1 and M2 polarization markers was also examined. The relative expression of iNOs mRNA increased under the EV stimulus and significantly decreased after secondary stimulation with IL-4 plus IL-10. As expected, stimulation with IL-4 plus IL-10 did not induce relative expression of iNOs, a scenario that was modified under re-stimulation with EVs, which promoted a significant relative expression of iNOs mRNA (Fig. 4c). Further, the relative expression of M2 polarization markers (Arginase and YM1) was induced by IL-4 plus IL-10 and not by EVs. The expression induced by IL-4 plus IL-10 decreased significantly under restimulation with EVs (Fig. 4c,d), whereas restimulation with IL-4 plus IL-10 reverted the negative response to EV-stimulation.

**Figure 5:**
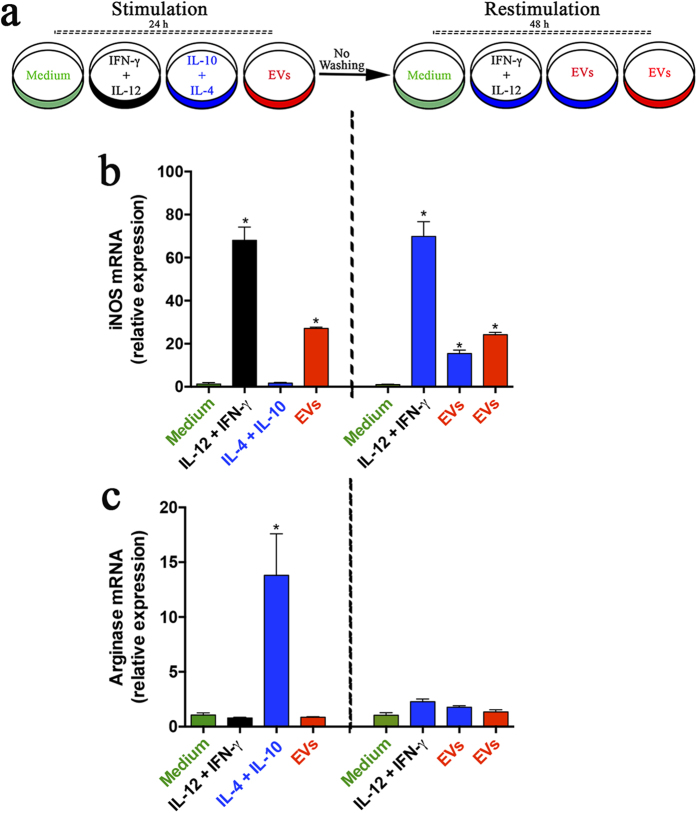
EVs from *P. brasiliensis* induce M1 macrophages under conditions that favor the development of M2 phenotype. **(a)** Peritoneal macrophages (2 × 10^6^/mL) were incubated at 37 °C with EVs (10 *μ*g/mL; red), IL-4 plus IL-10 (50 ng/mL both; blue), IL-12 plus IFN-γ (50 ng/mL both; black) and the medium as a negative control (green). After 24 h, the cells previously stimulated with IL-4 plus IL-10 (blue) were re-stimulated for 48 h with IL-12 plus IFN-γ (50 ng/mL both; black) or EVs (40 μg/mL; red). The cells were analyzed by real-time PCR for the relative expression of iNOS (**b**) and Arginase (**c**). The results, expressed as mean ± SEM, were compared to those obtained from the negative control (medium, green). Differences were considered significant when p < 0.05 (*).

In addition, we stimulated murine peritoneal macrophages with EVs (10 μg/mL), IL-4 (50 ng/mL) plus IL-10 (50 ng/mL), or IL-12 (50 ng/mL) plus IFN-γ (50 ng/mL). After 24 h of maintained stimulation to develop M2 macrophages, cells were re-stimulated, for 48 h, with EVs or IL-12 plus IFN-γ (Fig. 5a). The relative expression of iNOs (M1) and Arginase (M2) was determined, showing that iNOs mRNA increased after re-stimulation with EV or IL-12 plus IFN-γ (Fig. 5b). In contrast, re-stimulation with EVs did not change the Arginase expression by macrophages, i.e., it was similar to the verified in the absence of re-stimulus (medium) (Fig. 5c), indicating that the M2 phenotype of macrophages was maintained. The major conclusion of these results is that *P. brasiliensis* EVs can stimulate macrophage switching from an M2 toward an M1 phenotype.

### EVs from *P. brasiliensis* stimulate the fungicidal activity of macrophages

**Figure 6:**
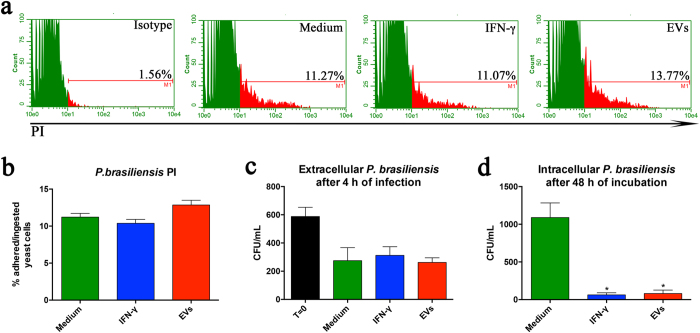
EVs from *P. brasiliensis* enhanced the fungicidal activity of macrophages. Peritoneal macrophages (1.5 × 10^6^/mL), treated for 24 h with EVs (10 μg/mL), IFN-γ (50 ng/mL), or the medium were washed and infected with *P. brasiliensis* (yeast-to-macrophage ratio of 1:1) and labeled with propidium iodide (PI), for 2 h. The macrophages were analyzed by flow cytometry. The percentage of PI-stained cells is shown in (**a**) (histogram) and (**b**) (bars). Alternatively, macrophages were infected with *P. brasiliensis* (yeast-to-macrophage ratio of 1:10), for 4 h. The cell supernatants were harvested and assessed for CFU, as a measurement of the presence of extracellular *P. brasiliensis* (**c**). After washing gently, the medium was added to the cells, and they were cultured for an additional 48 h. The cells were washed and lysed for the detection of viable intracellular yeasts by CFU (**d**). The results, expressed as mean ± SEM, were compared to those obtained from the negative control. Differences were considered significant when p < 0.05 (*).

Because EV-stimulation of macrophages induces the production of proinflammatory mediators and M1 polarization, we examined whether EVs could promote phagocytosis and killing of *P. brasiliensis*. Macrophages previously treated for 24 h with IFN-γ (50 ng/mL), EVs (10 μg/mL), or the medium were incubated with propidium iodide (PI)-stained *P. brasiliensis*. After 2 h, the percentage of macrophages containing adhering or internalized PI-stained yeasts was determined by flow cytometry. A similar proportion of macrophages containing PI-stained yeasts was found in unstimulated cells and cells stimulated with IFN-γ or EVs (Fig. 6a). In addition, the CFU recovery from the supernatant of murine peritoneal macrophages was similar between unstimulated, IFN-γ-stimulated, and EVs-stimulated cells (Fig. 6b). Remarkably, after 48 h of incubation, the CFU recovery from the macrophage lysate showed that the stimulation with either IFN-γ or EVs was associated with a CFU count lower than that obtained from unstimulated macrophages (Fig. 6d). We conclude that EV-stimulated macrophages exhibit a high fungicidal activity, comparable to those induced by IFN γ.

## Discussion

Herein we reported that EVs from *P. brasiliensis* induce the activation of peritoneal macrophages and that they can modulate the host innate immunity. We verified that murine peritoneal macrophages and the murine macrophage cell line J774A.1 released pro-inflammatory mediators in response to EVs stimulation, which in turn accounted for the development of M1 macrophages. Moreover, we showed that EVs could repolarize M2 macrophages toward an M1 profile, which was accompanied by an enhanced fungicidal capacity of the macrophages.

EVs released from infected cells have been studied more often than those released by the pathogens themselves. The studies found that EVs of host origin may contain inflammatory mediators that recruit and activate leukocytes and promote elimination of the invading organism. Nonetheless, the EVs released from infected cells contain pathogen components, many of which are known to act as major drivers of host immunity [reviewed by ref. 33]. The presence of EVs released from infected cells was demonstrated during viral, bacterial, parasitical, and fungal infections, and their primary function was shown to be immunomodulation. In the present study, we focused on vesicles released directly by the fungi, an approach we adopted from prior studies showing that extracellular vesicles from *Cryptococcus neoformans* activate macrophages, promoting cytokine production and restricting the fungal infection^15^. Furthermore, another study showed that *C. neoformans* EVs favor the *C. neoformans* infection, since interference with the export of exosomes decreased the virulence of *C. neoformans*^11^. A recent study demonstrated that EVs from *Malassezia sympodialis* enhance TNF-α and IL-4 production in peripheral blood mononuclear cells^34^, whereas EVs from *Candida albicans* stimulate macrophages and dendritic cells to produce proinflammatory mediators and up-regulate the expression of CD86 and MHC-II^9^. In addition, EVs isolated from supernatants of *Saccharomyces cerevisae* and *Histoplasma capsulatum* have been well characterized^13^,^15^; however, the studies provided limited insight into the influence of their EVs on immunomodulation. Regarding the studies on EVs from *P. brasiliensis*, Puccia and colleagues performed a series of studies that provide relevant information regarding the characterization of EVs. They elucidated the molecules of *P. brasiliensis* that EVs carry into the extracellular environment^16^ by describing the proteome^17^, lipidome^18^, as well as the RNA^19^ and carbohydrate^20^ content of the vesicles. However, the influence of *P. brasiliensis* EVs on the host immunity and PCM pathogenesis were not investigated.

*P. brasiliensis* EVs stimulated the development of M1 macrophages, which was characterized by the production of inflammatory cytokines and nitric oxide and by the augmented expression of iNOS2, besides reinforced by the fact that *P. brasiliensis* EVs did not induce macrophages to produce IL-10 (data not show). This profile contrasted with the one of M2 macrophages, induced by IL-4 plus IL-10 in our study, which was discriminated by the expression of Arginase-1, FIZZ1, and YM-1^35^. Immune modulation towards the M1 phenotype is strongly correlated to the outcome of the immune response to fungal infections, contributing to fungal clearance during experimental infections with *Cryptococcus neoformans*^32^ and *Candida albicans*^36^,^37^,^38^. However, there are somewhat conflicting observations on the role played by M1 or M2 macrophages in *P. brasiliensis* infection. The susceptible B10A mouse strain has an innate tendency to polarize macrophages into M1 phenotype, and the opposite polarization occurs in resistant A/J mice, supporting the idea that susceptibility and resistance to *P. brasiliensis* infection are strongly associated with M1 and M2 polarization, respectively^39^,^40^,^41^. However, the association of resistance with M1 polarization is supported by the fact that dectin-1 KO mice infected with *P. brasiliensis* develop M2 polarization, which impairs the fungicidal ability and diminishes survival rates (27). The association of M1 polarization and resistance to *P. brasiliensis* infection was reinforced recently by the report that the fungal lectin paracoccin, which exerts a therapeutic effect against PCM^42^, induces M1 polarization of peritoneal macrophages^43^.

We found that EVs from *P. brasiliensis* induce macrophages to switch their phenotype from M2 to M1. Interestingly, during the course of *C. neoformans* infection, the polarization status of pulmonary macrophages changes over time by repolarization of individual macrophages or the replacement of M2-polarized (non-protective) by new, M1-polarized (protective) cells. These mechanisms support the long-term persistence of the pathogen^32^. In addition, *C. albicans* blocked switching of the M2 to the M1 phenotype through the suppression of the host inflammatory response^44^. The plasticity of macrophage polarization during *P. brasiliensis* infection has not been previously evaluated; our data demonstrate, for the first time, the occurrence of macrophage repolarization from M2 to M1 in response to *P. brasiliensis* EVs. Furthermore, we showed that such M1 polarization is only partially reverted by the presence of M2-driving cytokines. Although we did not assess the occurrence of macrophage polarization *in vivo*, we hypothesize that *P. brasiliensis* EVs modulate immunity of infected mice. Ongoing studies of our laboratory investigate whether the intranasal administration of *P. brasiliensis* EVs effectively activates alveolar macrophages, as verified *in vitro*, and induces resistance to the infection. Positive responses may open new perspectives in the immunotherapy of PCM.

We used PI-stained *P. brasiliensis* to evaluate the interaction of the yeast with macrophages that were previously stimulated with EVs. The M1 polarization of macrophages showed to be induced by EVs and did not influence phagocytizing of the yeasts. The proportion of macrophages that adhered to or ingested PI-stained yeasts was similar between cells incubated with EVs, IFN-γ, or the medium alone. The responses to IFN-γ and the medium were close to those reported by Costa *et al*.^31^ when studying the influence of IL-10 on the phagocytic activity of macrophages. The results we obtained in the phagocytosis assay were consistent with the number of CFUs recovered from the extracellular milieu, which did not differ among macrophages incubated with EVs, IFN-γ, or the medium alone either. However, as reported by Oliveira *et al*.^15^, EVs from *C. neoformans* enhanced the phagocytic activity of RAW 264.7.

Notably, macrophages stimulated with EVs showed enhanced intracellular killing of *P. brasiliensis*, evident by the lower recovery of yeast CFU from lysed macrophages. This finding is consistent with the fact that the production of NO, a fungicidal compound, increased following macrophage stimulation with EVs from *P. brasiliensis*, as well as with IFN-γ. EVs from *C. neoformans* and *C. albicans* were also reported to enhance the fungicidal activity of macrophages^9^,^15^.

**Figure 7:**
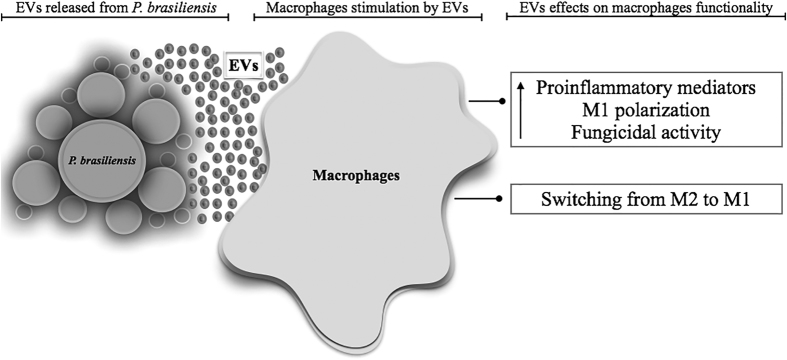
A proposed model that demonstrate the effects of EVs from *P. brasiliensis* on murine peritoneal macrophages. EVs from *P. brasiliensis* yeast have the ability to induce proinflammatory mediator’s production by murine peritoneal macrophages. Moreover, M1 polarization induced by EVs is associated with the increase in fungicidal activity. In addition, EVs from *P. brasiliensis* induce switching from M2 to M1 macrophages, a fact that suggests the EVs activity as a modulator of innate immune response.

In summary, we studied the ability of EVs from *P. brasiliensis* to modulate peritoneal macrophage polarization with an effector mechanism of fungal elimination. In this context, EV components from *P. brasiliensis* account for macrophage activation, production of pro-inflammatory mediators, M1 polarization, and killing of the fungus. These biological activities triggered by EVs on peritoneal macrophages were summarized in a proposed model shown in Fig. 7. The effects of *P. brasiliensis* EVs on macrophage functionality raise questions regarding the benefits of the host or the immune response subversion eventually provided by the production of EVs.

## Methods

### Ethics statement

This study was approved by the Committee of Ethics in Animal Research of the College of Medicine of Ribeirão Preto at the University of São Paulo and was conducted in accordance with the Ethical Principles in Animal Research adopted by the Brazilian College of Animal Experimentation, Protocol number 20/2013-1.

### Mice and *P. brasiliensis* isolates

Male C57BL/6 (wild-type, WT) mice at 6–8 weeks of age were used in this study. They were acquired from the animal house of the Campus of Ribeirão Preto, University of São Paulo, Ribeirão Preto, São Paulo, Brazil. The animals were housed in the animal facility of the Molecular and Cellular Biology Department of the Faculty of Medicine of Ribeirão Preto, University of São Paulo, under optimized hygienic conditions.

The *P. brasiliensis* isolate Pb18 was maintained by weekly subcultivation in the YPD medium (2% peptone, 1% yeast extract, and 2% glucose) in the yeast phase, at 36 °C. To ensure virulence of the yeast, serial passages in BALB/c mice were performed before the isolate Pb18 was used in experiments.

### Vesicle isolation

EVs were obtained as previously described by Vallejo *et al*.^16^. Yeast cells were transferred into Erlenmeyer flasks containing 200 mL of Ham’s F12 medium (Gibco, Carlsbad, CA, USA) supplemented with 1.5% glucose (Sigma-Aldrich, St. Louis, MO, USA) and cultivated for 4 days at 36 °C with continuous shaking (pre-inocula). The yeast cells from the pre-inocula were harvested and inoculated in 500 mL of fresh medium for another 48 h. A cell-free supernatant was obtained by sequential centrifugation at 5,000 *g* (15 min) and 15,000 *g* (30 min) at 4 °C. The pellets were discarded, and the supernatants ultra/diafiltrered using a membrane with a 100-kDa cutoff (Millipore, Billerica, MA, USA). The material was centrifuged at 15,000 *g* (30 min) to remove aggregates, and the resulting supernatant was centrifuged at 100,000 *g* for 1 h to precipitate vesicles. Vesicle pellets were washed in phosphate-buffered saline (PBS) and finally suspended in PBS for further analysis. The vesicle quantification was performed based on the analysis of sterol in their membranes, using a quantitative fluorimetric Amplex Red sterol assay kit (Molecular Probes, Thermo Fisher Scientific, Waltham, MA, USA), according to the manufacturer’s instructions.

### Preparation of murine peritoneal macrophages and the murine macrophage cell line J774A.1

Peritoneal macrophages were obtained from the peritoneal cavity of C57BL/6 mice that received an intraperitoneal injection of 1.0 mL of 3% sterile sodium thioglycollate medium (Sigma-Aldrich) 4 days earlier. The cells were harvested by peritoneal lavage with 5 mL of cold sterile PBS and centrifuged at 300 *g* (10 min at 4 °C). The pellet obtained was washed twice at 300 *g* for 10 min at 4 °C in Roswell Park Memorial Institute (RPMI) 1640 medium (Life Technologies, Grand Island, NY, USA) supplemented with 10% fetal cow serum (FCS; Life Technologies), 1% streptomycin/penicillin, and 2 mM L-glutamine (Sigma-Aldrich). Cells of the macrophage lineage J774A.1 were cultured in T75 flasks in RPMI 1640 containing 10% FCS supplemented with 1% streptomycin/penicillin and 2 mM L-glutamine (Sigma-Aldrich). Flasks were kept at 37 °C in an atmosphere of humidified air containing 5% CO_2_. The cells were maintained at subconfluent densities, and passages were performed every 2–3 days.

Macrophages and macrophage-like cells were incubated with EVs at different concentrations (1.25–40 μg/mL), LPS (1 μg/mL) plus IFN-γ (2 ng/mL), IL-4 (50 ng/mL) plus IL-10 (50 ng/mL), IFN-γ (50 ng/mL) plus IL-12 (50 ng/mL), or with the medium alone. The macrophages were cultured for 6 h for real-time quantitative PCR analysis, 48 h for determining the TNF-α, IL-12p40, IL-12p70, IL-1β, IL-1α, IL-6, and NO levels, or 24 h for quantifying their phagocytosis and killing of *P. brasiliensis*.

### Measurement of nitric oxide (NO) production

Supernatants from macrophage cell cultures (1.5 × 10^6^/mL; 7.5 × 10^5^ cells/well; 48-well plates) were used to measure the production of NO, which was inferred by measuring the accumulation of nitrite in the monolayer supernatants by a standard Griess reaction^45^. In short, 50 μL of each supernatant was incubated with an equal volume of Griess reagent (1.0% sulfanilamide, 0.1% naphthalenediaminedihydrochloride, and 2.5% H_3_PO_4_) and distributed in a 96-well microplate for 10 min at room temperature. The absorbance at 550 nm was measured using a Power Wave-X microplate reader (BioTek Instruments, Inc., Winooski, VT, USA). The conversion of absorbance values into micromolar (μM) concentrations of NO was done on the basis of a standard curve, concomitantly generated by using known concentrations of NaNO_2_ diluted in RPMI medium.

### Cytokine measurement

Supernatants of stimulated macrophages (1.5 × 10^6^/mL; 7.5 × 10^5^ cells/well; 48-well plates) were assessed for their levels of IL-12p40, IL-12p70, IL-6, TNF-α, IL-1α, and IL-1β. The cytokines were measured by capture enzyme-linked immunosorbent assay (ELISA) with antibody pairs purchased from BD Biosciences (Pharmingen, San Diego, CA, USA). The ELISA was performed according to the manufacturer’s protocol. The cytokine concentrations were determined from standard curves, using murine recombinant cytokines as a standard. The absorbance was read at 450 nm in the Power Wave-X microplate scanning spectrophotometer (BioTek Instruments, Inc.).

### Quantitative reverse transcription (qRT)-PCR

After 6 h of stimulation, the murine peritoneal macrophages (2 × 10^6^/mL; 1 × 10^6^ cells/well; 24-well plates) were used to extract the total RNA using the Trizol Reagent (Invitrogen, Life Technologies, Camarillo, CA, USA), according to the manufacturer’s instructions. The total RNA was reverse-transcribed into cDNA by the ImProm-II Reverse Transcription System (Promega, Fitchburg, WI, USA) using oligo(dT). qRT-PCR was performed in 15 μL, the reactions were using carried out by SYBR Green (Applied Biosystems/Life Technologies, Carlsbad, CA, USA) on a 7500 Real-Time PCR System (Applied Biosystems), under the following conditions: 50 °C for 2 min, 95 °C for 10 min, and 40 cycles of 95 °C for 15 sec/60 °C for 1 min. The relative expression of transcripts was quantified using the ΔΔCt method, and β-actin was used as the endogenous control. PCR primers utilized were: β-actin (F-CCTAAGGCCAACCGTGAAAA, R-GAGGCATACAGGGACAGCACA), Ym1 (F-TCACAGGTCTGGCAATTCTTCTG, R-ACTCCCTTCTATTGGCCTGTCC), Arginase-1 (F-GTTCCCAGATGTACCAGGATTC, R-CGATGTCTTTGGCAGATATGC), FIZZ1 (F-CCTGAGATTCTGCCCCAGGAT, R-TTCACTGGGACCATCAGCTGG), and iNOS2 (F-CCGAAGCAAACATCACATTCA, R-GGTCTAAAGGCTCCGGGCT).

### Phagocytosis and fungicidal activity assays of macrophages

Thioglycollate-induced peritoneal macrophages (1.5 × 10^6^/mL; 7.5 × 10^5^ cells/well; 48-well plates) were infected with *P. brasiliensis* cells for phagocytosis and fungicidal activity assays 24 h after stimulation. For the phagocytosis assay, the infection was performed with propidium iodide (PI)-stained *P. brasiliensis*, at a macrophage-to-yeast ratio of 1:1. The labeling of *P. brasiliensis* cells was performed according to the method reported by other studies^31^,^40^,^46^. The cells were cocultivated for 2 h at 37 °C. The supernatants were then removed and the cells washed with PBS to remove any yeast cells that were not ingested or that did not adhere. The macrophages were harvested and analyzed by flow cytometry (Guava easyCyte, Guava Technologies, Millipore, Hayward, CA, USA) for PI-staining. For the fungicidal activity assay, macrophages were cocultivated with *P. brasiliensis* in a macrophage-to-yeast ratio of 10:1 for 4 h at 37 °C. The macrophage monolayer was then washed, and the macrophages were cultivated for an additional 48 hours. Afterwards, the supernatants were removed and distilled water (100 μL) was added to the monolayer to promote macrophage lysis. The material was assayed for the presence of viable yeasts by the colony forming units (CFU) assay, as described previously^47^.

### Statistical Analysis

Statistical analyses were performed using the *GraphPad Prism* software (GraphPad Software, San Diego, CA). All data are presented as the mean ± SEM (standard error mean) of independent experiments performed (n = 3 for each group). Statistical determinations of the difference between means of groups were performed with the analysis of variance (1-way), followed by Bonferroni’s multiple comparison tests. Differences with p < 0.05 were considered statistically significant.

## Additional Information

**How to cite this article**: da Silva, T. A. *et al*. Extracellular vesicles from *Paracoccidioides brasiliensis* induce M1 polarization *in vitro*. *Sci. Rep.* **6**, 35867; doi: 10.1038/srep35867 (2016).
